# Description of three new *Peptoniphilus* species cultured in the vaginal fluid of a woman diagnosed with bacterial vaginosis: *Peptoniphilus pacaensis* sp. nov., *Peptoniphilus raoultii* sp. nov., and *Peptoniphilus vaginalis* sp. nov.

**DOI:** 10.1002/mbo3.661

**Published:** 2018-06-21

**Authors:** Khoudia Diop, Awa Diop, Caroline Michelle, Magali Richez, Jaishriram Rathored, Florence Bretelle, Pierre‐Edouard Fournier, Florence Fenollar

**Affiliations:** ^1^ Aix Marseille Univ, IRD, AP‐HM, SSA, VITROME IHU‐Méditerranée Infection Marseille France; ^2^ Aix‐Marseille Univ, IRD, AP‐HM, MEPHI, IHU‐Méditerranée Infection Marseille France; ^3^ Department of Gynecology and Obstetrics Gynépole, Hôpital Nord, AP‐HM Marseille France

**Keywords:** bacterial vaginosis, culturomics, human microbiota, *Peptoniphilus pacaensis*, *Peptoniphilus raoultii*, *Peptoniphilus vaginalis*, taxogenomics

## Abstract

Three previously unidentified Gram‐positive anaerobic coccoid bacteria, strains KhD‐2^T^, KHD4^T^, and Kh‐D5^T^, isolated from a vaginal swab, were characterized using the taxonogenomics concept. The phylogenic analysis, phenotypic characteristics, and genotypic data presented in this report attest that these three bacteria are distinct from previously known bacterial species with standing in nomenclature and represent three new *Peptoniphilus* species. Strain KhD‐2^T^ is most closely related to *Peptoniphilus* sp. DNF00840 and *Peptoniphilus harei* (99.7% and 98.2% identity, respectively); strain KHD4^T^ to *Peptoniphilus lacrimalis* (96%) and strain Kh‐D5^T^ to *Peptoniphilus coxii* (97.2%). Strains KhD‐2^T^, KHD4^T^, and Kh‐D5^T^ DNA G+C contents are, respectively, 34.23%, 31.87%, and 49.38%; their major fatty acid was C_16:0_ (41.6%, 32.0%, and 36.4%, respectively). We propose that strains KhD‐2^T^ (=CSUR P0125 = DSM 101742), KHD4^T^ (=CSUR P0110 = CECT 9308), and Kh‐D5^T^ (=CSUR P2271 = DSM 101839) be the type strains of the new species for which the names *Peptoniphilus vaginalis* sp. nov.*, Peptoniphilus raoultii* sp. nov., and *Peptoniphilu pacaensis* sp. nov., are proposed, respectively.

## 1 INTRODUCTION

Since the 1800s, physicians and researchers investigate the vaginal bacterial community using both cultivation and culture‐independent methods (Pandya et al., 2017; Srinivasan et al., 2016). To date, many species from the vaginal microbiota have been identified. The healthy vaginal flora is associated to a biotope rich in *Lactobacilli* species (Li, McCormick, Bocking, & Reid, 2012). The vaginal microbiota has a beneficial relationship with its host and can also impact women's health, that of their partners as well as their neonates (Lepargneur & Rousseau, 2002; Srinivasan & Fredricks, 2008). A depletion of vaginal *Lactobacilli* can lead to bacterial vaginosis (BV). This disease is a dysbiosis that may be associated to sexually transmitted infections as well as miscarriage and preterm birth in pregnant women (Afolabi, Moses, & Oduyebo, 2016; Martin & Marrazzo, 2016).

A microbial culturomics study exploring the bacterial community of the vaginal econiche flora in healthy women and patients suffering from bacterial vaginosis enabled the isolation of three Gram‐positive‐staining, anaerobic, and coccoid bacteria in the vaginal discharge of a woman with bacterial vaginosis (Lagier et al., 2015, 2016). These bacteria exhibited phylogenetic and phenotypic proximity to species of the *Peptoniphilus* genus. Created after the division of *Peptostreptococcus* genus into five genera (Ezaki et al., 2001), the *Peptoniphilus* genus belonging to the Peptoniphilaceae family that regroup members of the genera *Peptoniphilus*,* Parvimonas, Murdochiella*,* Helcococcus*,* Gallicola*,* Finegoldia, Ezakiella*,* Anaerosphaera*, and *Anaerococcus* (Johnson, Whitehead, Cotta, Rhoades, & Lawson, 2014; Patel et al., 2015). The *Peptoniphilus* genus is currently made of 16 valid published species (http://www.bacterio.net/peptoniphilus.html ). These bacteria employ amino acids and peptone as a major energy sources (Ezaki et al., 2001). They are mainly cultivated from diverse human samples such as sacral ulcer, vaginal discharge, as well as ovarian, peritoneal, and lacrymal gland abscesses (Ezaki et al., 2001; Li et al., 1992; Ulger‐Toprak, Lawson, Summanen, O'Neal, & Finegold, 2012).

Herein, we describe the isolation and taxonogenomic characterization (Fournier, Lagier, Dubourg, & Raoult, 2015) of strains KhD‐2^T^, KHD4^T^, and Kh‐D5^T^ as type strains of three new *Peptoniphilus* species for which the names *Peptoniphilus vaginalis* sp. nov. (=CSUR P0125, =DSM 101742)*, Peptoniphilus raoultii* sp. nov. (=CSUR P0110, =CECT 9308), and *Peptoniphilus pacaensis* sp. nov. (=CSUR P2271, =DSM 101839), are proposed, respectively. All the three strains were cultivated from the vaginal swab of the same patient.

## 2 MATERIALS AND METHODS

### 2.1 Samples and ethics

The vaginal specimen from a French 33‐year‐old woman with bacterial vaginosis was sampled at Hospital Nord in Marseille (France) in October 2015 using a Sigma Transwab (Medical Wire, Corsham, United Kingdom). Bacterial vaginosis was diagnosed as previously described (Menard, Fenollar, Henry, Bretelle, & Raoult, 2008). The patient had not received any antibiotic for several months. The local IFR48 ethics committee in Marseille (France) authorized the study (agreement number: 09‐022). In addition, the patient gave her signed informed consent.

### 2.2 Bacterial strain isolation and identification

After sampling, the specimen was preincubated in a blood culture bottle (Becton‐Dickinson Diagnostics, Le Pont‐de‐Claix, France). The blood culture bottle was enriched with 3 ml of sheep blood (bioMérieux, Marcy l'Etoile, France) and 4 ml of rumen fluid, filter‐sterilized through a 0.2 μm pore filter (Thermo Fisher Scientific, Villebon‐sur‐Yvette, France). Various preincubation periods (1, 3, 7, 10, 15, 20, and 30 days) were tested. Then, 50 μl of the supernatant were inoculated on both Colistin‐nalidixic acid (CNA) used for selective enrichment of Gram‐positive bacteria and trypticase soy agar plates used for cultivation of nonfastidious and fastidious microorganisms (both BD Diagnostics), and then incubated for 4 days under anaerobic conditions at 37°C. Isolated colonies were purified and subsequently identified by matrix‐assisted laser‐desorption/ionization time‐of‐flight (MALDI‐TOF) mass spectrometry with a Microflex spectrometer (Bruker, Leipzig, Germany) that compared the new spectra with those present in the library (Bruker database and URMITE database, constantly updated), as previously reported (Seng et al., 2009). If the score was >1.99, the bacterium was considered as identified at the genus level (score between 2.0 and 2.299) or species level (score from 2.3 to 3.0). When the score was <1.7, no identification was considered reliable. The 16S rRNA sequence of unidentified isolates was obtained using an ABI Prism 3130xl Genetic Analyzer capillary sequencer (Applied Biosystems, Bedford, MA, USA), as previously described (Morel et al., 2015; Seng et al., 2009). Finally, the sequences were compared to the NCBI nr database using the BLAST algorithm (https://blast.ncbi.nlm.nih.gov/Blast.cgi). If the 16S rRNA sequence similarity value was lower than 98.7%, the isolate was considered as a putative new species (Kim, Oh, Park, & Chun, 2014; Stackebrandt & Ebers, 2006; Yarza et al., 2014).

### 2.3 Phylogenetic analysis

The 16S rRNA sequences of isolates not identified using mass spectrometry and those of members of the family Peptoniphilaceae with standing in nomenclature (downloaded from the nr database) were aligned using CLUSTALW (Thompson, Higgins, & Gibson, 1994) with default setting. The phylogenetic inferences were performed using both the neighbor‐joining and maximum‐likelihood methods with the software MEGA version 6 (Tamura, Stecher, Peterson, Filipski, & Kumar, 2013).

### 2.4 Phenotypic characteristics

For each new isolate, cell morphology was visualized using optical and electron microscopy. Oxidase, catalase, motility, sporulation tests, as well as Gram stain were performed as already reported (Murray, Baron, Jorgensen, Landry, & Pfaller, 2007). Cells were fixed for electron microscopy for at least 1 hour at 4°C with 2.5% glutaraldehyde in a 0.1 mol L^−1^ cacodylate buffer. One drop of cell suspension was deposited for about 5 min on a glow‐discharged formvar carbon film on 400‐mesh nickel grids (FCF400‐Ni, EMS). The grids were dried on a blotting paper. Then, the cells were negatively stained at room temperature for 10 s with a 1% ammonium molybdate solution in filtered water. Micrographs were obtained using a Tecnai G20 Cryo (FEI) transmission electron microscope operated at 200 keV.

In order to characterize the best growth conditions of each isolate, bacteria were inoculated on 5% sheep blood‐enriched Columbia agar (bioMérieux) incubated at various atmospheres (aerobic, anaerobic, and microaerophilic) and temperatures (56, 42, 37, 28, and 25°C) (Mishra, Lagier, Nguyen, Raoult, & Fournier, 2013). Several salinity (NaCl concentrations of 0%, 5%, 15%, and 45%) and pH (5, 6, 6.5, 7, and 8.5) conditions were also tested.

Biochemical analyses were realized using various strips (API ZYM, API 20A, and API 50CH) according to the manufacturer's instructions (bioMérieux) (Avguštin, Wallace, & Flint, 1997; Durand et al., 2017). The tests were performed in anaerobic chamber. The strips were incubated there for 4, 24, and 48 hr, respectively.

For the analysis of cellular fatty acid methyl ester (FAME), gas chromatography/mass spectrometry (GC/MS) was achieved. All three isolates were grown anaerobically at 37°C on 5% sheep blood‐enriched Columbia agar (bioMérieux). For each isolate, after 2 days of incubation, two aliquots with roughly 25–70 mg of bacterial biomass per tube were prepared. FAME preparation and GC/MS analyses were performed as already reported (Dione et al., 2016; Sasser, 2006). FAMEs were separated with an Elite 5‐MS column and monitored by MS (Clarus 500‐SQ 8 S, Perkin Elmer, Courtaboeuf, France). A spectral database search was done with MS Search 2.0 operated using the standard reference database 1A (NIST, Gaithersburg, USA) as well as the FAMEs mass spectral database (Wiley, Chichester, UK).

The susceptibility of all three isolates was tested for 11 antibiotics: amoxicillin (0.16–256 μg/ml), benzylpenicillin (0.002–32 μg/ml), ceftriaxone (0.002–32 μg/ml), ertapenem (0.002–32 μg/ml), imipenem (0.002–32 μg/ml), amikacin (0.16–256 μg/ml), erythromycin (0.16–256 μg/ml), metronidazole (0.16–256 μg/ml), ofloxacin (0.002–32 μg/ml), rifampicin (0.002–32 μg/ml), and vancomycin (0.16–256 μg/ml). Minimal inhibitory concentrations (MICs) were estimated using E‐test strips (bioMérieux) and according to EUCAST recommendations (Citron, Ostovari, Karlsson, & Goldstein, 1991; Matuschek, Brown, & Kahlmeter, 2014).

### 2.5 Genome sequencing and analyses

After a pretreatment of 2 hr at 37°C using lysozyme, the genomic DNAs (gDNAs) of strains KhD‐2^T^, KHD4^T^, and Kh‐D5^T^ were extracted using the EZ1 biorobot and EZ1 DNA Tissue kit (Qiagen). An elution volume of 50 μl was obtained for each sample. The gDNAs were quantified by a Qubit assay (Life technologies, Carlsbad, CA, USA) at 74.2, 22.4, and 16.4 ng/μl, respectively. Genomic sequencing of each strain was performed with a MiSeq sequencer (Illumina Inc, San Diego, CA, USA) and the Mate Pair strategy.

The Mate Pair library was prepared with the Nextera Mate Pair guide (Illumina) using 1.5 μg of gDNA. The gDNA samples were fragmented and tagged using a Mate Pair junction adapter (Illumina). Then, the fragmentation pattern was validated using a DNA 7500 labchip on an Agilent 2100 BioAnalyzer (Agilent Technologies Inc, Santa Clara, CA, USA). No size selection was done. Thus, 537, 600, and 480.7 ng of tagmented fragments were, respectively, circularized. Circularized DNAs were mechanically cut to smaller fragments using Optima on a bimodal curve at 507 and 1,244 bp for KhD‐2^T^, 975 and 1,514 bp for KHD4^T^, and 609 and 999 bp for Kh‐D5^T^ on the Covaris device S2 in T6 tubes (Covaris, Woburn, MA, USA). The libraries profiles were visualized on a High Sensitivity Bioanalyzer LabChip (Agilent Technologies Inc, Santa Clara, CA, USA) and the final concentrations libraries were determined. Then, the libraries were normalized at 2 nmol L^−1^, pooled, denatured, diluted at 15 pmol L^−1^, loaded onto the reagent cartridge, and onto the instrument. Sequencing was performed in a single 39‐hr run in a 2 × 250‐bp.

The genome assembly was performed with a pipeline that enabled to create an assembly with various software such as Velvet (Zerbino & Birney, 2008), Spades (Bankevich et al., 2012), and Soap Denovo (Luo et al., 2012), on trimmed data with MiSeq and Trimmomatic (Bolger, Lohse, & Usadel, 2014) software or untrimmed data with only MiSeq software. In order to reduce gaps, GapCloser was used (Luo et al., 2012). Phage contamination was searched (blastn against Phage Phix174 DNA sequence) and eliminated. Finally, scaffolds with sizes under 800 bp and scaffolds with a depth value lower than 25% of the mean depth were identified as possible contaminants and removed. The best assembly was considered by using several criteria including number of scaffolds, N50, and number of N. Spades gave the best assembly for the three studied strains with depth coverage of 518x.

Prodigal was used to predict open reading frames (ORFs) (Hyatt et al., 2010) using default parameters. However, the predicted ORFs were excluded if they spanned a sequencing gap region (containing Ns). The predicted bacterial protein sequences were analyzed as previously reported (Alou et al., 2017). tRNA genes were found using the tRNAScan‐SE tool (Lowe & Eddy, 1997), while RNAmmer was used to find ribosomal RNAs (Lagesen et al., 2007). Phobius was used to predict lipoprotein signal peptides and the number of transmembrane helices (Käll, Krogh, & Sonnhammer, 2004). ORFans were identified when the BLASTP search failed to provide positive results (*E*‐value smaller than 1e^−03^ for ORFs with a sequence size larger than 80 aa or an *E*‐value smaller than 1e^−05^ for ORFs with a sequence length smaller than 80 aa), as previously reported (Alou et al., 2017). For genomic comparison, the closest species with validly published names in the 16S RNA phylogenetic tree were identified with the Phylopattern software (Gouret, Thompson, & Pontarotti, 2009). The complete genome, proteome, and ORFeome sequences were retrieved for each selected species in NCBI. An annotation of the entire proteome in order to define the distribution of functional classes of predicted genes according to the COG classification of their predicted protein products was performed as already reported (Alou et al., 2017). Annotation and comparison processes were done using the DAGOBAH software as previously described (Alou et al., 2017; Gouret et al., 2005, 2011). Finally, in order to evaluate the genomic similarity between the genomes, we determined two previously described parameters: average amino acid identity (AAI) based on the overall similarity between two genomic datasets of proteins available at (http://enve-omics.ce.gatech.edu/aai/index) and digital DNA–DNA hybridization (dDDH) (Auch, von Jan, Klenk, & Göker, 2010; Meier‐Kolthoff, Auch, Klenk, & Göker, 2013; Alou et al., 2017; Rodriguez & Konstantinidis, 2014; Chun et al., 2018).

## 3 RESULTS

### 3.1 Strain identification and phylogenetic analysis

**Figure 1:**
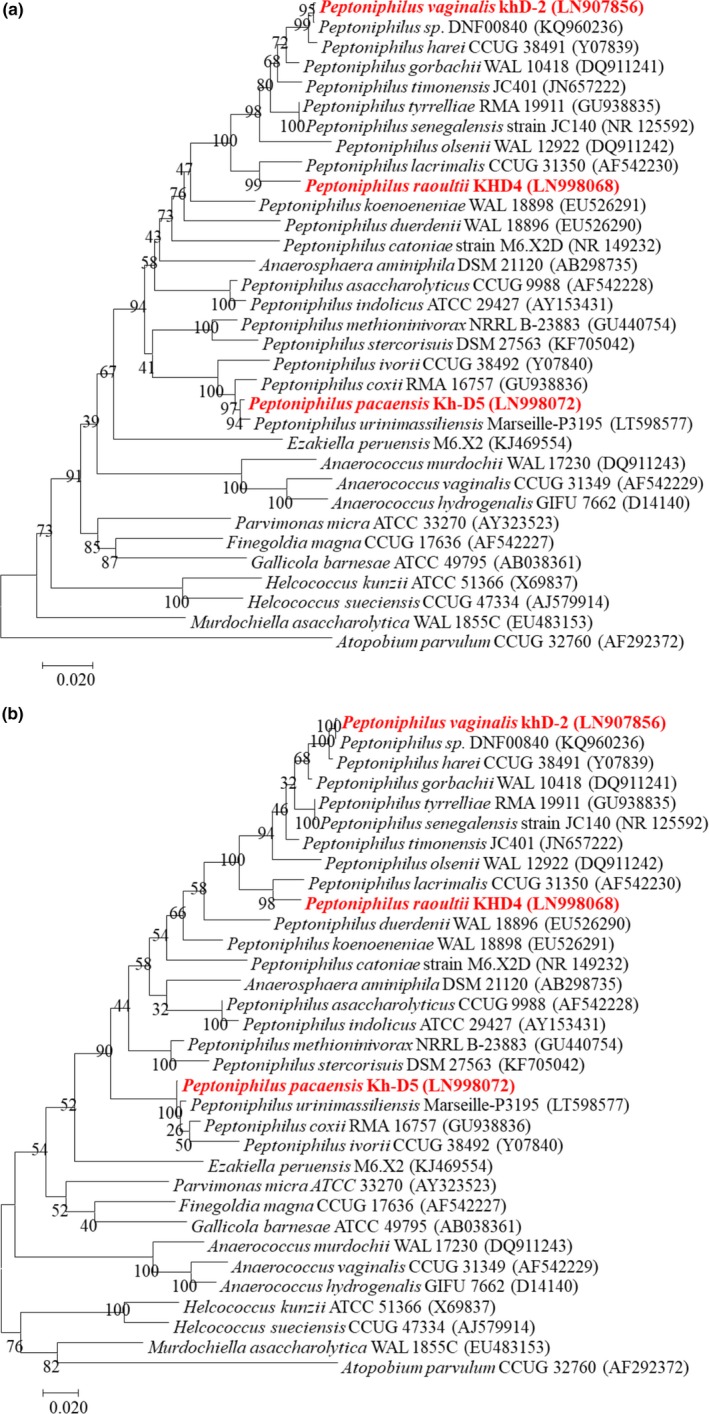
Phylogenetic analysis based on the 16S RNA gene sequence highlighting the position of *Peptoniphilus vaginalis* strain KhD‐2^T^, *Peptoniphilus raoultii* strain KHD4^T^, and *Peptoniphilus pacaensis* strain Kh‐D5^T^ relative to other closely related strains. GenBank accession numbers are indicated in parentheses. Sequences were aligned using Muscle v3.8.31 with default parameters and, phylogenetic inferences were performed using the neighbor‐joining (a) and maximum‐likelihood (b) methods with the software MEGA version 6. The scale bar represents a 2% nucleotide sequence divergence

The MS identification of the three bacteria, secluded, respectively, after 24 hr (strains KhD‐2^T^ and KHD4^T^) and 15 days (Kh‐D5^T^) of preincubation, failed. This suggested that these isolates were not in the database and may be unknown species. Pairwise analysis of 16S rRNA sequences attested that strain KhD‐2^T^ exhibited 92.8% and 87.4% sequence similarities with strains KHD4^T^ and Kh‐D5^T^, respectively, and strains KHD4^T^ and Kh‐D5^T^ had an 88.7% identity. BLASTN sequence searches demonstrated that the three strains were related to the genus *Peptoniphilus*, suggesting that each strain represented a new species within this genus. Strain KhD‐2^T^ exhibited a 16S rRNA similarity of 99.7% with *Peptoniphilus* sp. strain DNF00840 (GenBank KQ960236) over 1,842 bp and 98.2% with *Peptoniphilus harei* (GenBank NR_026358.1) over 1,488 bp. Strain KHD4^T^ exhibited a 16S rRNA similarity of 96% with *Peptoniphilus lacrimalis* (GenBank NR_041938.1) over 1,489 bp. Finally, strain Kh‐D5^T^ exhibited a 16S rRNA similarity of 97.2% with *Peptoniphilus coxii* (GenBank NR_117556.1) over 1,491 bp (Figure 1). As these percentage similarities were under the threshold of 98.7% established to delineate new species (Kim et al., 2014; Stackebrandt & Ebers, 2006; Yarza et al., 2014), strains KhD‐2^T^, KHD4^T^, and Kh‐D5^T^ were considered as representative strains of putative new *Peptoniphilus* species. The names *P. vaginalis* sp. nov., *P. raoultii* sp. nov., and *P. pacaensis* sp. nov. are, respectively, proposed.

**Figure 2:**
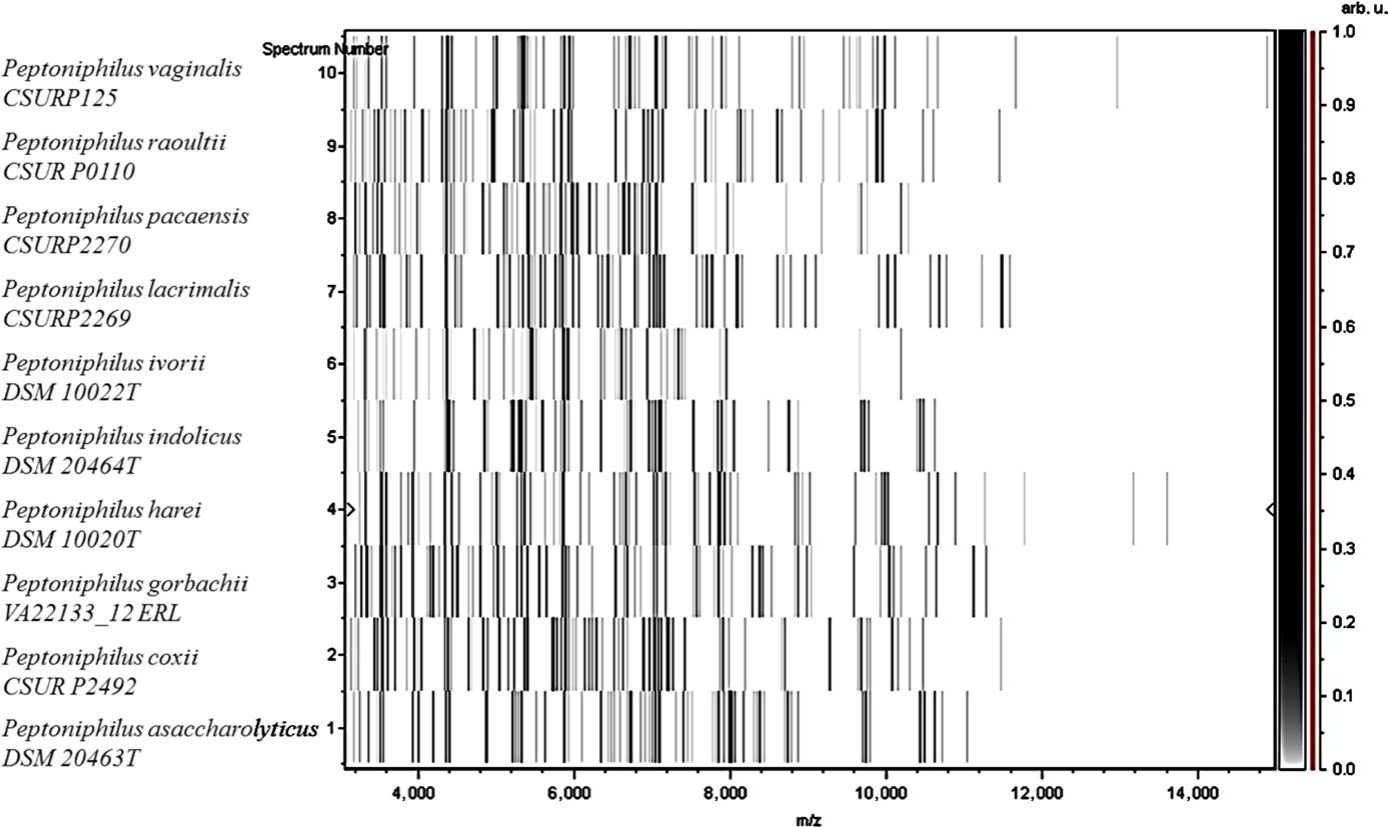
Gel view comparing strains KhD‐2^T^, KHD4^T^, and Kh‐D5^T^ to other species within the genus *Peptoniphilus*. The gel view displays the raw spectra of loaded spectrum files arranged in a pseudo‐gel‐like look. The *x*‐axis records the *m*/*z* value. The left *y*‐axis displays the running spectrum number originating from subsequent spectra loading. The peak intensity is expressed by a gray scale scheme code. The right *y*‐axis indicates the relation between the color of a peak and its intensity, in arbitrary units. Displayed species are indicated on the left

The reference MALDI‐TOF MS spectra of our isolates were added in our database (http://www.mediterranee-infection.com/article.php?laref=256&amp;titre=urms-database) and then compared to those of other *Peptoniphilus* spp. (Figure 2).

### 3.2 Phenotypic features

Cells from all three novel strains (KhD‐2^T^, KHD4^T^, and Kh‐D5^T^) were Gram‐ ‐positive cocci (mean diameter of 0.6–0.7 μm for each). After 4 days of incubation, colonies on blood agar were grey and circular, and all had a diameter ranging from 1 to 2 mm. For all the three strains, growth occurred only in anaerobic atmosphere. Besides, optimal growth occurred at 37°C, with a pH between 6.5 and 8.5, and a NaCl concentration lower than 5%. They exhibited no catalase, oxidase, and urease activities. Using API 20A strips, all tests including aesculin, arabinose, cellobiose, gelatin, glucose, glycerol, indole, lactose, maltose, mannitol, mannose, raffinose, rhamnose, saccharose, sorbitol, trehalose, urease, and xylose were negative for strains KHD4^T^ and Kh‐D5^T^, whereas for strain KhD‐2^T^, indole formation was positive, and gelatin was hydrolyzed. API ZYM strips showed that the three isolates exhibited positive reactions for acid phosphatase, esterase, and Naphthol‐AS‐BI‐phosphohydrolase. In addition, strains KhD‐2^T^ and KHD4^T^ had *N*‐acetyl‐β‐glucosaminidase and leucine arylamidase activities. In contrast, an alkaline phosphatase activity was observed for strains KhD‐2^T^ and Kh‐D5^T^. All other remaining tests including valine arylamidase, lipase, cystine arylamidase, trypsin, galactosidase, glucosidase, β‐glucuronidase, α‐mannosidase, and α‐fucosidase were negative. Using API 50CH strips, all three isolates fermented ribose, tagatose, and potassium‐5‐ketogluconate. However, they did not ferment adonitol, aesculin, arabinose, arabitol, cellobiose, dulcitol, erythritol, fructose, fucose, galactose, glucose, glycerol, glycogen, inulin, lyxose, inositol, mannose, mannitol, maltose, melibiose, potassium gluconate, potassium‐2‐ketogluconate, salicine, saccharose, sorbitol, sorbose, trehalose, melezitose, raffinose, rhamnose, starch, turanose, xylitol, and xylose. Table 1 displayed the phenotypic differences between these bacteria and other *Peptoniphilus* spp.

**Table 1:** **C**ompared phenotypic characteristics of *Peptoniphilus vaginalis* strain KhD‐2^T^, *Peptoniphilus raoultii* strain KHD4^T^, *Peptoniphilus pacaensis* strain Kh‐D5^T^, and other closely related *Peptoniphilus* species. Data were obtained from the original descriptions of species

Properties	*P. vaginalis*	*P. raoultii*	*P. pacaensis*	*P. harei*	*P. lacrimalis*	*P. coxii*	*P. duerdenii*	*P. indolicus*	*P. asaccharolyticus*
Cell diameter (μm)	0.66	0.7	0.7	0.5–1.5	0.5–0.7	<0.7	≥0.7	0.7–1.6	0.5–1.6
% G+C	34.23	31.87	49.38	34.44	30.22	44.62	34.24	31.69	32.30
Major fatty acid (%)	C_16:00_ (41.6)	C_16:00_ (32)	C_16:00_ (36.4)	C_16:00_ (31.2)	C_16:00_ (27.7)	C_16:00_ (49.9)	C_16:00_ (33)	C_16:00_ (19.4)	C18:2ω6 (22.0)
Production of									
Alkaline phosphatase	+	−	+	−	−	−	−	+	+
Indole	+	−	−	+	−	−	+	+	−
Catalase	−	−	−	+	na	−	−	−	−
Urease	−	−	−	−	−	−	−	−	−
β‐galactosidase	−	−	−	−	−	−	−	−	−
*N*‐Acetyl‐β‐glucosaminidase	+	+	−	na	na	−	−	na	na
Acid from									
Ribose	+	+	+	−	−	−	−	−	−
d‐fructose	+	−	−	−	−	−	−	−	−
Habitat	Human vagina	Human vagina	Human vagina	Human sacral ulcer	Human eyes	Human specimens	Human vagina	Summer mastitis of cattle	Human vagina

+, positive; −, negative; v, variable and na (not available) data.

The fatty acid composition of the three strains was as following: strain KhD‐2^T^ contained saturated acid C_16:0_ (41.6%) and C_14:0_ (14.7%); unsaturated acids were also detected (Table 2); strains KHD4^T^ and Kh‐D5^T^ contained C_16:0_ (32% and 36%, respectively), C_18:2ω6_ (26% and 24%, respectively), and C_18:1ω9_ (26% and 21%, respectively) (Table 2). These fatty acid results were likened to those of related species in Table 2 (Johnson et al., 2014; Rooney, Swezey, Pukall, Schumann, & Spring, 2011). Strain KhD‐2^T^ can be distinguished from its nearest neighbor *P. harei* by the production of C_14:0_ (14.7% vs. 4.4%). Strain KHD4^T^ can be distinguished from its closest related species *P. lacrimalis* by the presence of fatty acids: C_14:0_, C_17:0_ iso 3‐OH, and anteiso‐C_17:0_. Finally, strain Kh‐D5^T^ showed a fairly similar profile with its neighbors *P. coxii* and *Peptoniphilus ivorii* with some differences such as the presence of antesio‐C_5:0,_ only in strain Kh‐D5^T^ (4.5%), of iso‐C_5:0_ in *P. coxii* (5.5%), and C_17:0_ iso 3‐OH and antesio‐C_17:0_, solely in *P. ivorii* (7.7% and 3.8%, respectively). Besides, the three strains were sensitive to amoxicillin, benzylpenicillin, ceftriaxone, ertapenem, imipenem, metronidazole, rifampicin, and vancomycin, but resistant to amikacin, erythromycin, and ofloxacin (Table 3).

**Table 2:** Cellular fatty acid profiles (%) of strains KhD‐2^T^, KHD4^T^, and Kh‐D5^T^ compared with other *Peptoniphilus* species

Fatty acids	Name	1	2	3	4	5	6	7	8	9	10
C4:00	Butanoic acid	TR	−	−	−	−	−	−	−	−	−
iso‐C5:0	3‐Methyl‐butanoic acid	−	−	−	−	−	5.5	−	−	−	−
anteiso‐C5:0	2‐Methyl‐butanoic acid	TR	−	4.5	−	−	−	−	−	−	−
C10:0	Decanoic acid	−	−	TR	TR	−	−	2.8	TR	−	−
C12:0	Dodecanoic acid	TR	−	TR	−	TR	TR	−	1.2	TR	2.3
C13:0	Tridecanoic acid	TR	−	−	−	−	−	−	−	−	−
C14:0	Tetradecanoic acid	**14.7**	TR	4.9	4.4	2.9	8.6	4.4	**12.6**	4.4	5.4
C14:1ω5	9‐Tetradecenoic acid	TR	−	−	−	−	−	−	−	−	−
C15:0	Pentadecanoic acid	1.1	TR	TR	−	−	1.4	−	−	−	−
C16:0	Hexadecanoic acid	**41.6**	**32.0**	**36.4**	**32.1**	**27.7**	**49.9**	**33.0**	**19.4**	**29.5**	**14.4**
C16:0 9,10‐methylene	2‐Hexyl‐cyclopropaneoctanoic acid	−	TR	−	−	−	−	−	−	−	−
C16:1ω5	11‐Hexadecenoic acid	TR	−	−	−	−	−	−	−	−	−
C16:1ω7	9‐Hexadecenoic acid	6.2	1.0	TR	1.0	3.2	−	−	−	1.0	3.9
C16:1ω9	7‐Hexadecenoic acid	TR	−	−	−	−	−	−	3.6	−	−
C17:0	Heptadecanoic acid	TR	TR	TR	−	−	−	−	−	−	−
C17:0 iso 3‐OH	3‐Hydroxy‐heptadecanoic acid	−	−	−	6.0	3.0	−	−	−	7.7	‐
anteiso‐C17:0	14‐Methyl‐hexadecanoic acid	TR	−	−	4.2	1.8	−	−	2.6	3.8	1.6
C17:1ω7	10‐Heptadecenoic acid	TR	−	−	−	−	−	−	−	−	−
C18:0	Octadecanoic acid	3.9	8.8	3.6	7.2	**11.2**	**13.1**	**16.2**	2.5	4.8	9.4
C18:1ω7	11‐Octadecenoic acid	4.8	3.7	2.0	1.9	3.5	−	−	3.5	2.6	−
C18:1ω9	9‐Octadecenoic acid	**12.1**	**25.8**	**21.2**	**17.0**	**25.7**	**17.3**	**22.6**	6.2	**11.4**	**20.2**
C18:2ω6	9,12‐Octadecadienoic acid	**12.0**	**26.4**	**24.4**	**17.0**	**13.6**	3.2	**21.1**	**13.0**	**24.0**	**22.0**

Strains: 1, *P. vaginalis* strain KhD‐2^T^; 2, *P. raoultii* strain KHD4^T^; 3, *P. pacaensis* strain Kh‐D5^T^; 4, *Peptoniphilus harei* DSM 10020^T^; 5, *P. lacrimalis* DSM 7455^T^; 6, *P. coxii* CSUR 2492^T^; 7, *P. uerdenii* WAL 18896^T;^ 8, *P. indolicus* DSM 20464^T^, 9, *P. ivorii* CCUG 38492^T^ and 10, *P. asaccharolyticus* CCUG 9988^T^. Strains 1, 2, 3, and 6 data are from this study and strains 4, 5, 7 to 9, data come from Rooney et al., 2011 and Johnson et al., 2014. Predominant products are shown in bold; TR, trace amounts < 1%; −, not detected.

**Table 3:** Minimal inhibitory concentrations (MIC μg/μl) of antibiotics for *P. vaginalis* strain KhD‐2^T^, *P. raoultii* strain KHD4^T^, and *P. pacaensis* strain Kh‐D5^T^

Antibiotics	Concentration (μg/ml)	*P. vaginalis* strain KhD‐2^T^	*P. raoultii* strain KHD4^T^	*P. pacaensis* strain Kh‐D5^T^
Amoxicillin	0.016–256	0.032	0.016	0.016
Benzylpenicillin	0.002–32	0.094	0.002	0.002
Ceftriaxone	0.002–32	0.064	0.064	0.064
Ertapenem	0.002–32	0.002	0.003	0.002
Imipenem	0.002–32	0.004	0.002	0.002
Metronidazole	0.016–256	0.125	0.032	0.032
Rifampicin	0.002–32	0.002	0.002	0.002
Vancomycin	0.016–256	0.094	0.094	0.094
Amikacin	0.016–256	>256	>256	>256
Erythromycin	0.016–256	1	2	2
Ofloxacin	0.002–32	>256	>256	2

### 3.3 Genome characteristics

**Figure 3:**
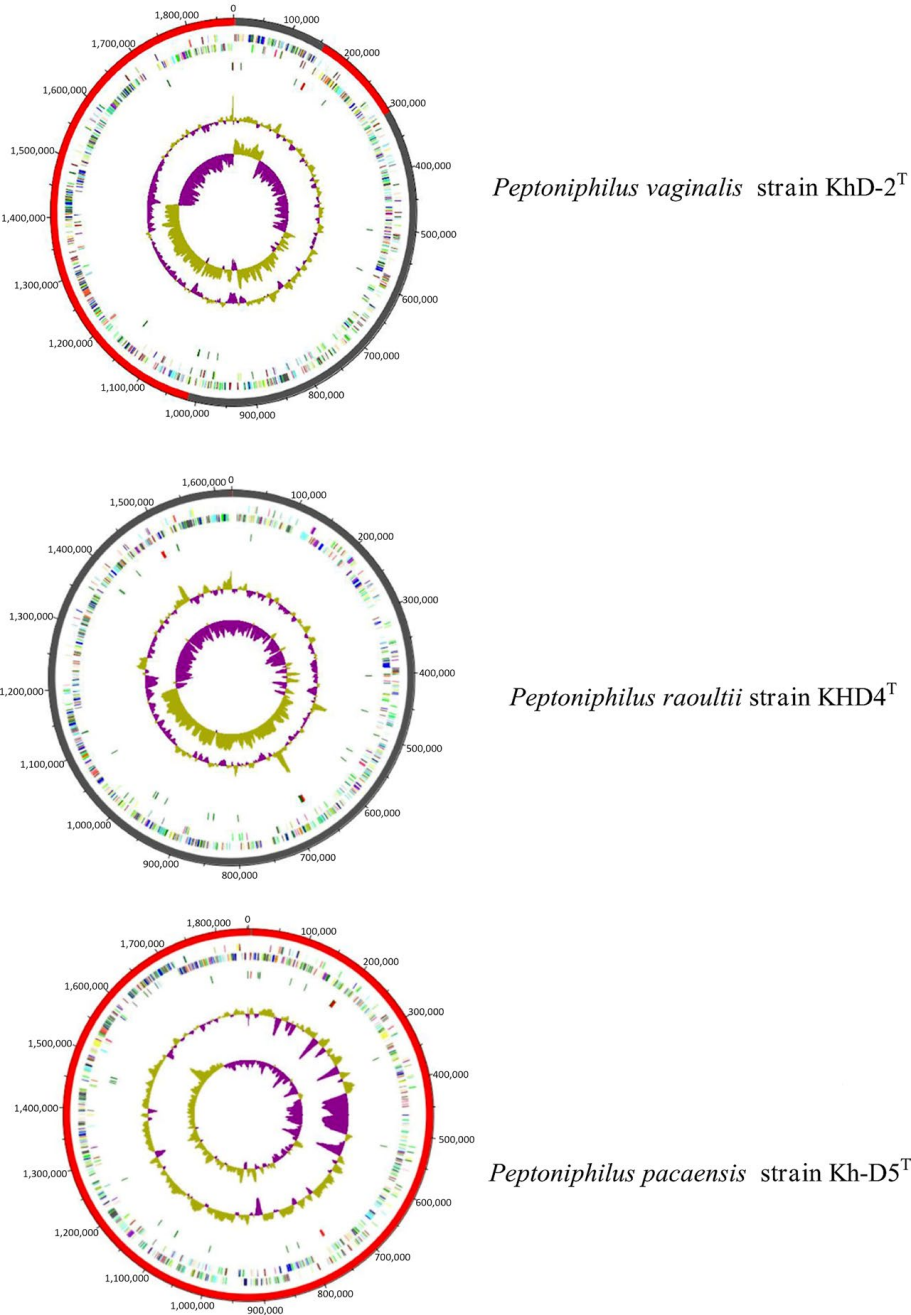
Graphical circular map of the three genomes. From outside to the center: Contigs (red/gray), COG category of genes on the forward strand (three circles), genes on forward strand (blue circle), genes on the reverse strand (red circle), COG category on the reverse strand (three circles), G+C content

**Figure 4:**
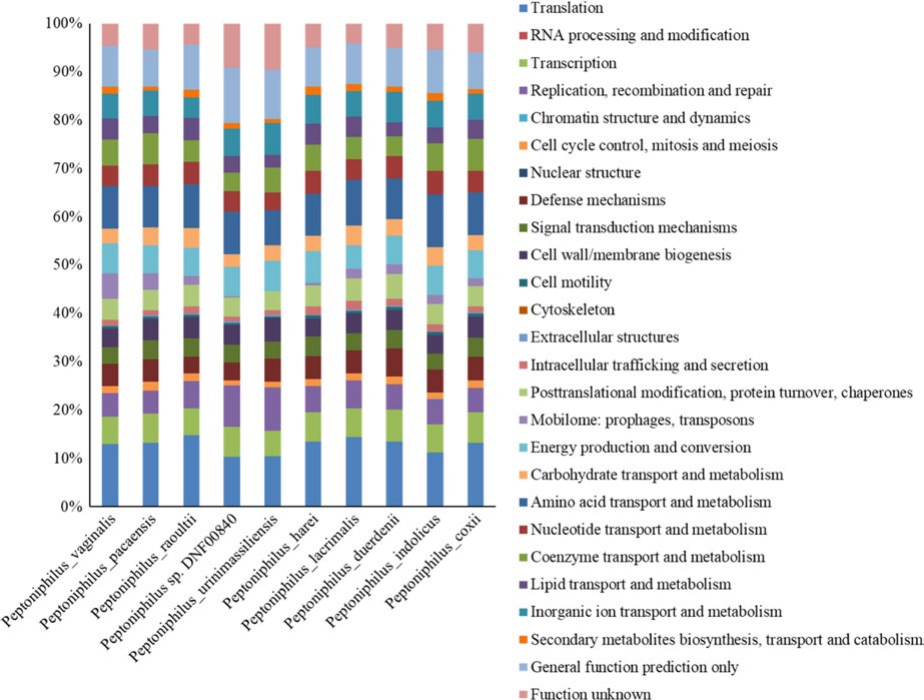
Distribution of functional classes of predicted genes according to the clusters of orthologous groups of proteins of *P. vaginalis* strain KhD‐2^T^, *P. raoultii* strain KHD4^T^, and *P. pacaensis* strain Kh‐D5^T^ among other species

**Figure 5:**
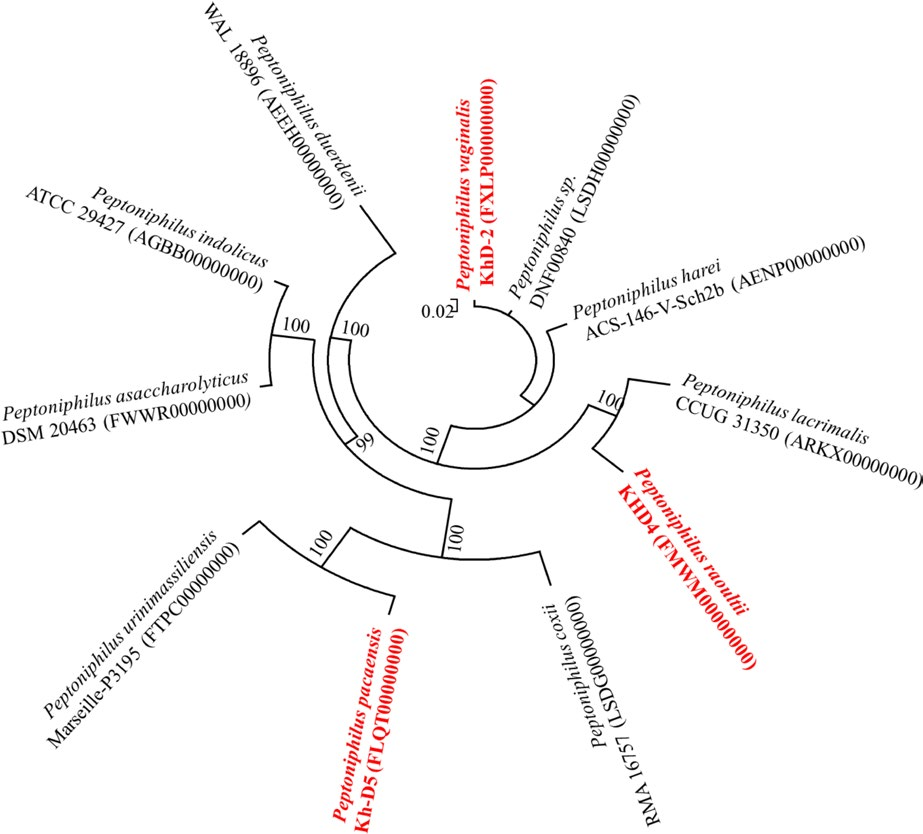
Phylogenetic tree based on whole genome sequence showing the position of *P. vaginalis* strain KhD‐2^T^, *P. raoultii* strain KHD4^T^, and *P. pacaensis* strain Kh‐D5^T^ relative to their nearest neighbors. GenBank accession numbers are indicated in parentheses. Sequences were aligned using Mugsy software, and phylogenetic inferences were performed using the maximum likelihood method with the software FastTree. The scale bar represents a 2% nucleotide sequence divergence

Strains KhD‐2^T^, KHD4^T^, and Kh‐D5^T^ exhibited genomes sizes of 1,877,211, 1,623,601, and 1,851,572 bp long, respectively (Figure 3). The genome characteristics were detailed in Table 4. The repartition of genes into the 25 general COG categories was represented in Table 5 and Figure 4. When compared to other *Peptoniphilus* species, the three strains had genome sizes, G+C contents and total gene counts in the same range (Table 6, Figure 5). Although, base composition varies widely among bacterial species, the genes within a given genome are relatively similar in G+C content with the exception of recently acquired genes. As a matter of fact, DNA sequences acquired by horizontal transfer often bear unusual sequence characteristics and can be distinguished from ancestral DNA notably by a distinct G+C content (Lawrence & Ochman, 1997). The region between 100,000 and 600,000 bp of the chromosome from strain KhD‐5^T^ showed a high variation in G+C content (Figure 3). Thus, 43 genes putatively acquired by horizontal gene transfer were identified in this region, including 25 genes specific for strain KhD‐5^T^ and 18 genes shared with strain *Peptoniphilus urinimassiliensis*. Consequently, the presence of these genes may play a role in the significant difference in genomic G+C content observed between strain KhD‐5^T^ and other compared *Peptoniphilus* species as well as the similar genomic G+C content observed between strain KhD‐5^T^ and *P. urinimassiliensis*.

**Table 4:** Nucleotide and gene count levels of the genomes

	*P. raoultii*		*P. vaginalis*		*P. vaginalis*	
Attribute	Value	% of total^a^	Value	% of total^a^	Value	% of total^a^
Size (bp)	1,623,601	100%	1,877,211	100%	1,851,572	100%
G+C content (bp)	517,506	31.87%	642,534	34.22%	914,357	49.38%
Coding region (bp)	1,467,557	90.39%	1,692,527	90.16	3,579,496	85.07%
Total genes	1,624	100%	1,780	100%	1,801	100%
RNA genes	42	2.59%	40	2.35%	54	3.00%
Protein‐coding genes	1,520	93.60%	1,698	95.39%	1,699	94.34%
Genes with function prediction	1,222	75.25%	1,375	77.24%	1,323	73.45%
Genes assigned to COGs	1,048	65.53%	1,204	67.64%	1,175	65.24%
Genes with peptide signals	162	9.97%	169	9.49%	231	12.83%
Genes with transmembrane helices	349	21.49%	403	22.64%	414	22.98%

a The total is based on either the size of the genome in base pairs or the total number of protein coding genes in the annotated genome.

**Table 5:** Number of genes associated with the 25 general COG functional categories

	*P. vaginalis*		*P. raoultii*		*P. pacaensis*		
Code	Value	% value	Value	% value	Value	% value	Description
J	170	9.70	170	10.69	171	9.78	Translation
A	0	0	0	0	0	0	RNA processing and modification
K	75	4.28	63	3.96	78	4.46	Transcription
L	64	3.65	65	4.09	63	3.60	Replication, recombination, and repair
B	0	0	0	0	0	0	Chromatin structure and dynamics
D	20	1.14	18	1.13	23	1.31	Cell cycle control, mitosis, and meiosis
Y	0	0	0	0	0	0	Nuclear structure
V	61	3.48	40	2.51	60	2.97	Defense mechanisms
T	44	2.51	43	2.70	52	3.64	Signal transduction mechanisms
M	50	2.85	50	3.14	55	3.14	Cell wall/membrane biogenesis
N	7	0.39	7	0.44	8	0.45	Cell motility
Z	0	0	0	0	0	0	Cytoskeleton
W	3	0.17	3	0.18	2	0.11	Extracellular structures
U	15	0.85	16	1.00	15	0.85	Intracellular trafficking and secretion
O	58	3.31	51	3.20	54	3.08	Posttranslational modification, protein turnover, chaperones
X	68	3.88	22	1.38	44	2.51	Mobilome: prophages, transposons
C	83	4.74	66	4.15	75	4.29	Energy production and conversion
G	40	2.28	47	2.95	48	2.74	Carbohydrate transport and metabolism
E	115	6.56	105	6.60	112	6.40	Amino acid transport and metabolism
F	57	3.25	52	3.27	58	3.31	Nucleotide transport and metabolism
H	71	4.05	52	3.27	84	4.80	Coenzyme transport and metabolism
I	56	3.19	53	3.33	45	2.57	Lipid transport and metabolism
P	68	3.88	48	3.02	69	3.94	Inorganic ion transport and metabolism
Q	19	1.08	18	1.13	11	0.62	Secondary metabolites biosynthesis, transport, and catabolism
R	111	6.33	107	6.73	98	5.60	General function prediction only
S	62	3.54	51	3.20	71	4.06	Function unknown
‐	547	31.23	541	34.04	573	32.78	Not in COGs

**Table 6:** Genome comparison of closely related species to *P. vaginalis* strain KhD‐2^T^, *P. raoultii* strain KHD4^T^, and *P. pacaensis* strain Kh‐D5^T^

Species	INSDC identifier^a^	Size (Mbp)	G+C Percent	Gene Content	Number of contigs	N50 Value
***P. vaginalis*** **KhD‐2** ^**T**^	**FXLP00000000**	**1.88**	**34.2**	**1,791**	**5**	**707,77**
***P. raoultii*** **KHD4** ^**T**^	**FMWM00000000**	**1.62**	**31.9**	**1,631**	**2**	**1,62**
***P. pacaensis*** **Kh‐D5** ^**T**^	**FLQT00000000**	**1.85**	**49.4**	**1,802**	**3**	**1,84**
*Peptoniphilus* sp. DNF00840	LSDH00000000	1.88	34.3	1,671	91	50,04
*Peptoniphilus urinimassiliensis* Marseille‐P3195	FTPC00000000	1.82	49.7	1,770	5	563,37
*Peptoniphilus harei* ACS‐146‐V‐Sch2b	AENP00000000	1.84	34.4	1,749	32	111,2
*Peptoniphilus lacrimalis* CCUG 31350	ARKX00000000	1.85	30.2	1,785	22	190,04
*Peptoniphilus duerdenii* WAL 18896	AEEH00000000	2.12	34.2	1,963	61	96,77
*Peptoniphilus indolicus* ATCC 29427	AGBB00000000	2.24	31.7	2,145	302	11,79
*Peptoniphilus coxii* RMA 16757	LSDG00000000	1.84	44.6	1,783	48	103,89
*Peptoniphilus asaccharolyticus* DSM 20463	FWWR00000000	2.23	32.3	2,054	17	1,358,172

a INSDC: International Nucleotide Sequence Database Collaboration. Text and values ​​in bold have been used to highlight new species.

The dDDH values ranked from 20.1% ± 2.3% between *P. harei* and *P. duerdenii* to 56.4% ± 2.75% between *P. lacrimalis* and *P. urinimassiliensis* (Table 7). When comparing the three new strains to other *Peptoniphilus* species, strain KhD‐2^T^ exhibited dDDH values ranging from 22.7% ± 2.4% with *Peptoniphilus indolicus* to 47.3% ± 2.55% with *P. coxii*; dDDH values from strain KHD4^T^ ranged from 19.0% ± 2.25% with *P. harei* to 44.3% ± 2.55% with *P. coxii*; and strain Kh‐D5^T^ exhibited dDDH values ranging from 20.7% ± 2.35% with *P. coxii* to 45.0% ± 2.60% with *P. urinimassiliensis* (Table 7). Furthermore, the AAI values ranged from 51.3% between *P. coxii* and *P. indolicus* to 84.0% between *P. indolicus* and *Peptoniphilus asaccharolyticus* (Table 8). Comparing the three new isolates to their neighbors, strain KhD‐2^T^ shared AAI values ranging from 51.5% with *P. urinimassiliensis* to 92.9% with *P. harei*, AAI values of strain KHD4^T^ ranging from 50.9% with *P. urinimassiliensis* to 70.6% with *P. lacrimalis*, and strain Kh‐D5^T^ exhibited AAI values ranging from 50.2% with *P. asaccharolyticus* to 92.9% with *P. urinimassiliensis* (Table 8). According to the fact that the threshold of dDDH and AAI values for distinguishing different species are 70% and 95%–96%, respectively (Chun et al., 2018; Klappenbach et al., 2007; Meier‐Kolthoff et al., 2013; Richter & Rosselló‐Móra, 2009; Rodriguez‐R & Konstantinidis, 2014), these data confirm the classification of strains KhD‐2^T^, KHD4^T^, and Kh‐D5^T^ in distinct species.

**Table 7:** dDDH values obtained by comparison of all studied genomes using GGDC, Formula 2 (DDH Estimates Based on Identities/HSP length)^a^

	*P. vaginalis* strain KhD‐2^T^	*P. raoultii* strain KHD4^T^	*P. pacaensis* strain Kh‐D5^T^	*P. urini‐massiliensis*	*P. harei*	*P. lacrimalis*	*P. duerdenii*	*P. indolicus*	*P. coxii*	*P. asaccharolyticus*
*P. vaginalis*	100 ± 00	22.9 ± 2.35	40.0 ± 2.50	35.3 ± 2.50	45.8 ± 2.60	25.6 ± 2.40	32.0 ± 2.45	22.7 ± 2.40	47.3 ± 2.55	33.20 ± 2.45
*P. raoultii*		100 ± 00	29.8 ± 2.45	40.5 ± 2.50	19.0 ± 2.25	20.4 ± 2.30	36.4 ± 2.55	22.2 ± 2.35	44.3 ± 2.55	28.40 ± 2.45
*P. pacaensis*			100 ± 00	45.0 ± 2.60	42.0 ± 2.55	41.9 ± 2.55	38.7 ± 2.50	27.3 ± 2.45	20.7 ± 2.35	29.30 ± 2.45
*P. urinimassiliensis*				100 ± 00	32.9 ± 2.50	56.4 ± 2.75	42.9 ± 2.50	33.0 ± 2.45	20.1 ± 2.30	32.30 ± 2.45
*P. harei*					100 ± 00	34.3 ± 2.50	39.2 ± 2.50	20.1 ± 2.30	36.2 ± 2.45	33.30 ± 2.45
*P. lacrimalis*						100 ± 00	39.3 ± 2.50	25.1 ± 2.40	40.6 ± 2.50	31.90 ± 2.45
*P. duerdenii*							100 ± 00	24.3 ± 2.35	38.2 ± 2.50	32.80 ± 2.50
*P. indolicus*								100 ± 00	44.0 ± 2.55	26.70 ± 2.45
*P. coxii*									100 ± 00	35.40 ± 2.45
*P. asaccharolyticus*										100 ± 00

a The confidence intervals indicate the inherent uncertainty in estimating DDH values from intergenomic distances based on models derived from empirical test data sets (which are always limited in size).

**Table 8:** AAI values obtained by comparison of all studied genomes

	*P. raoultii* strain KHD4^T^	*P. pacaensis* strain Kh‐D5^T^	*P. urini‐massiliensis*	*P. harei*	*P. lacrimalis*	*P. duerdenii*	*P. indolicus*	*P. coxii*	*P. asaccharolyticus*
*P. vaginalis*	62.7	51.2	51.5	92.9	61.5	57.0	55.9	53.2	57.9
*P. raoultii*		50.0	50.9	61.6	70.6	56.2	55.4	52.5	56.8
*P. pacaensis*			92.9	51.8	51.2	51.8	50.4	74.1	50.2
*P. urinimassiliensis*				52.0	52.7	52.2	51.4	73.4	51.3
*P. harei*					64.2	58.5	56.4	51.7	58.5
*P. lacrimalis*						58.0	55.9	51.8	57.1
*P. duerdenii*							54.7	53.1	57.0
*P. indolicus*								51.3	84.0
*P. coxii*									51.2

## 4 DISCUSSION

The aim of this study was to investigate, using culturomics, the vaginal flora of a woman with bacterial vaginosis. Indeed, bacterial vaginosis is a gynecologic disorder marked by a perturbation of the vaginal microbiota equilibrium with a loss of commensal *Lactobacillus* spp. and their replacement with anaerobic bacteria including *Atopobium vaginae*,* Bacteroides* spp., *Mobiluncus* spp., *Prevotella* spp., and numerous Gram‐positive anaerobic cocci (Bradshaw et al., 2006; Onderdonk, Delaney, & Fichorova, 2016; Shipitsyna et al., 2013). Gram‐positive anaerobic cocci were associated to various infections (Murdoch, 1998). They represent about 24%–31% of anaerobic bacteria cultivated in clinical specimens (Murdoch, Mitchelmore, & Tabaqchali, 1994). In this present study, three novel Gram‐positive‐staining, anaerobic cocci (KhD‐2^T^, KHD4^T^, and Kh‐D5^T^) were cultured in the vaginal discharge of a patient suffering from bacterial vaginosis. These bacteria exhibited sufficient MALDI‐TOF MS profiles, 16S rRNA sequence, phenotypic, and genomic differences with *Peptoniphilus* species to be regarded as representative strains of three new species within this genus. Currently, this genus contains 16 species with validly published names. Most of them have been observed in human clinical specimens (Ezaki et al., 2001).

Data from phylogenetic analysis and genomic comparison exhibited the heterogeneity of this genus and revealed that strain KhD‐2^T^ and *Peptoniphilus* sp. DNF00840^T^ share 99.79% 16S rRNA gene sequence similarity, an ANI value of 96.83% and 75.0% of dDDH. In fact, to differentiate bacterial species, thresholds lower than 98.7%, 94%, and 70% were delimited for 16S rRNA sequence identity, ANI, and dDDH values, respectively. Therefore, the obtained values suggest that the two strains (KhD‐2^T^ and *Peptoniphilus* sp. DNF00840^T^) belong to the same species. Unlike other *Peptoniphilus* spp., strains KhD‐2^T^, KHD4^T^, and Kh‐D5^T^ ferment ribose and tagatose. The study of their genomes revealed that strain Kh‐D2^T^ had 75 genes associated to carbohydrate metabolism, including 4 genes (1 *rbsA* gene, 2 *rbsR* genes, and 1 *rpiB* gene) encoding proteins involved in fermentation of ribose; the genome from strain KHD4^T^ contained 61 genes associated to carbohydrate metabolism of which one *rpiB* gene is involved in fermentation of ribose; and strain KhD‐5^T^ had 58 genes associated to carbohydrate metabolism with 3 genes implicated in ribose fermentation (2 *rpiB* genes and 1 *rbsK*) and 1 gene encoding a tagatose biphosphate aldolase enzyme involved in tagatose fermentation. In addition, the genomes of strains Kh‐D2^T^, KHD4^T^, and KhD‐5^T^ also had 25 genes (5 genes encoding proteins responsible for the degradation of histidine, 1 of lysine, 2 of threonine, 12 of methionine, and 5 of arginine), 20 genes (5 of histidine, 1 of lysine, 1 of threonine, 7 of methionine, and 6 of arginine), and 21 genes (14 which degraded methionine, 6 for arginine and 1 for lysine), associated to amino acid degradation, respectively.

Finally, we propose that strains KhD‐2^T^, KHD4^T^, and Kh‐D5^T^ are type strains of *P. vaginalis* sp. nov., *P. raoultii* sp. nov., and *P. pacaensis* sp. nov., respectively.

### 4.1 Description of *P. vaginalis* sp. nov

*Peptoniphilus vaginalis* (va.gi.na'lis. L. n. fem. gen. *vaginalis* from the feminine organ vagina; vaginalis pertaining to the vagina).

Gram‐stain—positive. Coccus‐shaped bacterium with a mean diameter of 0.66 μm. *Peptoniphilus vaginalis* sp. nov. is a mesophilic bacterium; its optimal growth occurs at temperature 37°C, a pH ranking from 6.5 to 8.5, and a NaCl concentration lower than 5%. Colonies are circular, translucent, gray, and have a diameter of 1–1.5 mm on Columbia agar. Cells are strictly anaerobic, not motile, and non‐spore‐forming. Catalase, oxidase, and urease activities are negative. Nitrate reduction is also negative nevertheless indole production is positive. *P. vaginalis* shows positive enzymatic activities for acid phosphatase, alkaline phosphatase, esterase, esterase lipase, leucine arylamidase, Naphthol‐AS‐BI‐phosphohydrolase, and *N*‐acetyl‐β‐glucosaminidase. *P. vaginalis* ferments fructose, potassium 5‐ketogluconate, ribose, and tagatose. C_16:0_, C_14:0_, C_18:1ω9_, and C_18:2ω6_ are its main fatty acids. Strain KhD‐2^T^ is sensitive to amoxicillin, benzylpenicillin, ceftriaxone, imipenem, ertapenem, metronidazole, rifampicin, and vancomycin but resistant to amikacin, erythromycin, and ofloxacin. Its 1,623,601‐bp genome contains 34.23% G+C. In EMBL‐EBI, the 16S rRNA gene sequence is deposited under accession number LN907856 and the draft genome sequence under accession number FXLP00000000. The type strain of *Peptoniphilus vaginalis* sp. nov. is strain KhD‐2^T^ (=CSUR P0125 = DSM 101742), which was cultured from the vaginal discharge of a woman suffering from bacterial vaginosis.

### 4.2 Description of *P. raoultii* sp. nov

*Peptoniphilus raoultii* (ra.oul'ti.i. N. L. masc. gen. n. *raoultii* of Raoult, to honor French scientist Professor Didier Raoult for his outstanding contribution to medical microbiology).

Gram‐stain—positive. Coccus‐shaped bacterium with a mean diameter of 0.7 μm. *Peptoniphilus raoultii* sp. nov. is a mesophilic bacterium; its optimal growth occurs at temperature 37°C, a pH ranking from 6.5 to 8.5, and a NaCl concentration lower than 5%. Colonies are circular, translucent, gray, and have a diameter of 1–1.5 mm on Columbia agar. Cells are strictly anaerobic, not motile, and non‐spore‐forming. Catalase, oxidase, urease, indole, and nitrate activities are negative. *P. raoultii* exhibits positive enzymatic activities for acid phosphatase, esterase, esterase lipase, leucine arylamidase, Naphthol‐AS‐BI‐phosphohydrolase, and *N*‐acetyl‐β‐glucosaminidase. *P. raoultii* ferments potassium 5‐ketogluconate, ribose, and tagatose. C_16:0_, C_18:2ω6_, and C_18:1ω9_ are its main fatty acids. Strain KHD4^T^ is sensitive to amoxicillin, benzylpenicillin, ceftriaxone, imipenem, ertapenem, metronidazole, rifampicin, and vancomycin but resistant to amikacin, erythromycin, and ofloxacin. The genome is 1,877,211 bp long and contains 31.87% G+C. In EMBL‐EBI, the 16S rRNA gene sequence is deposited under accession number LN998068 and the draft genome sequence under accession number FMWM00000000. Strain KHD4^T^ (=CSUR P0110 = CECT 9308) is the type strain of *P. raoultii* sp. nov., which was cultured from the vaginal discharge of a woman suffering from bacterial vaginosis.

### 4.3 Description of *P. pacaensis* sp. nov

*Peptoniphilus pacaensis* (pa.ca.en'sis N. L. gen. masc. n. *pacaensis*, from the acronym PACA, of Provence‐Alpes‐Côte d'Azur*,* the region where the type strain was first cultured and characterized).

Gram‐stain—positive. Coccus‐shaped bacterium with a mean diameter of 0.7 μm. *Peptoniphilus pacaensis* sp. nov. is a mesophilic bacterium; its optimal growth occurs at temperature 37°C, a pH ranking from 6.5 to 8.5, and a NaCl concentration lower than 5%. Colonies are circular, translucent, gray, and have a diameter of 1–1.5 mm on Columbia agar. Cells are strictly anaerobic, not motile, and non‐spore‐forming. Catalase, oxidase, urease, indole, and nitrate activities are negative. *P. pacaensis* shows positive enzymatic activities for alkaline phosphatase, acid phosphatase, esterase, esterase lipase, and Naphthol‐AS‐BI‐phosphohydrolase. *P. pacaensis* ferments potassium 5‐ketogluconate, ribose, and tagatose. C_16:0_, C_18:2ω6_, and C_18:1ω9_ are its main fatty acids. Strain Kh‐D5^T^ is sensitive to amoxicillin, benzylpenicillin, ceftriaxone, imipenem, ertapenem, metronidazole, rifampicin, and vancomycin but resistant to amikacin, erythromycin, and ofloxacin. Its genome is 1,851,572 bp long with a 49.38% G+C content. In EMBL‐EBI, the 16S rRNA gene sequence is deposited under accession number LN998072 and the draft genome sequence under accession number FLQT00000000. The type strain of *P. pacaensis* sp. nov. is strain Kh‐D5^T^ (=CSUR P2270 = DSM 101839), which was cultured from the vaginal discharge of a woman suffering from bacterial vaginosis.

## CONFLICT OF INTEREST

The authors declare no conflict of interest.
